# Long Non-Coding RNAs Involved in Immune Responses

**DOI:** 10.3389/fimmu.2014.00573

**Published:** 2014-11-13

**Authors:** Katsutoshi Imamura, Nobuyoshi Akimitsu

**Affiliations:** ^1^Radioisotope Centre, The University of Tokyo, Tokyo, Japan

**Keywords:** long non-coding RNA (lncRNA), innate immune response, NEAT1, translational repression

## Abstract

A large number of human RNA transcripts, which do not encode proteins are defined as non-coding RNAs (ncRNAs). These ncRNAs are divided into two classes of different lengths; short and long ncRNAs. MicroRNAs are a major class of short ncRNAs, ~22 nucleotides in length that regulate gene expression at the post-transcriptional level. Long non-coding RNAs (lncRNAs) are more than 200 nucleotides in length and play roles in various biological pathways. In this review, we summarize the functions of lncRNAs which regulate immune responses.

## Introduction

Whole transcriptome analyses of mammalian genomes, such as studies documented in the ENCODE project, have characterized RNA transcripts that have low-protein coding potential, known as non-coding RNAs (ncRNAs) (1). In recent years, it has become increasingly apparent that ncRNAs are involved in diverse biological processes (2–5). Based on their length, ncRNAs are classified into short ncRNAs and long non-coding RNAs (lncRNAs) (6). MicroRNAs (miRNAs) are a major class of short ncRNAs, ~22 nucleotides (nt) in length that regulate post-transcriptional gene silencing by controlling translation and RNA stability (7, 8). miRNAs are involved in the regulation of diverse biological processes, such as proliferation, differentiation, apoptosis, development, and immune responses (9–12). LncRNAs are longer than 200 nt and play roles in diverse biological processes, such as proliferation, differentiation, and development through various modes of action. For instance, several lncRNAs, such as XIST, ANRIL, and HOTAIR, recruit polycomb-repressive complex 2 to target loci for epigenetic regulation (13–18). Recently, it was reported that several lncRNAs regulate post-transcriptional gene regulation through binding to specific RNA-binding proteins (16).

The innate and adaptive immune responses provide immunity to pathogens. The innate immune response is the first line of defense. Cells infected with a pathogen trigger the innate immune response by synthesizing inflammatory mediators or cytokines through the transcriptional and post-transcriptional gene regulations. The innate immune response also activates adaptive immune responses resulting in facilitated elimination of the pathogen. Therefore, the innate immune response is crucial to the host for protection against pathogens, such as bacterium or viruses. A primary challenge for the innate immune system is the discrimination of pathogens by specific receptors. Toll-like receptors (TLRs) are a major receptor type for the recognition of pathogens (19). For example, TLR2 recognizes lipo-protein, TLR3 recognizes double-strand RNAs produced by viruses, TLR4 recognizes lipopolysaccharide, and TLR5 recognizes flagella of bacteria. Following ligand recognition by these receptors, specific signaling cascades are activated that results in the synthesis of inflammatory mediators or cytokines. This involves transcriptional or post-transcriptional gene regulation via transcription factors, such as API and NF-κB, and miRNAs (19, 20). For instance, miR-203 regulates inflammatory cytokines tumor necrosis factor (TNF)-α and interleukin (IL)-24 (21), while miR-26a, -34a, -145, and let-7b regulate IFN-β (22). However, the regulation of inflammatory mediators or cytokines by lncRNAs is still poorly understood. Several hypotheses have recently emerged concerning lncRNA involvement in infectious diseases (23) and some studies found that several lncRNAs might be involved in the regulation of immune responses. In this review, we focus on five lncRNAs, which are clearly related with immune responses. Three of them are involved in bacterial infection, because of stimulation of TLR2 or 4. Two of them are involved in viral infection.

## lncRNAs Involved in Bacterial Infection

### lincRNA-Cox2

Whole transcriptome analysis (RNA-seq) of mouse bone marrow-derived macrophages (BMDMs) showed that stimulation by the synthetic bacterial lipopeptide Pam_3_CSK_4_, a TLR2 ligand, increased the expression of 62 lncRNAs (24). In these lncRNAs, one of the most highly induced lncRNAs was lincRNA-Cox2 (ENSMUSG00000091113, Gm17311). Lipopolysaccharide, a TLR4 ligand, and R848, a synthetic antiviral compound that activates Tlr7/8, also increased lincRNA-Cox2 levels via the Myd88-NF-κB pathway. Conversely, polyinosinic-polycytidylic acid (poly I:C), a synthetic double-stranded RNA that is recognized by TLR3, did not affect lincRNA-Cox2 expression. LincRNA-Cox2 has three variants and is about 2 kb in length. LincRNA-Cox2 variant 1 is the most abundant transcript, but it is unclear, which one acts in immune response and these differences. LincRNA-Cox2 was localized in both the nuclear and the cytosolic compartments of macrophages.

In Pam_3_CSK_4_ treatment of BMDMs, silencing of lincRNA-Cox2 induced up-regulation of Ccl5, but down-regulation of Il-6. These results suggest that lincRNA-Cox2 mediates both activation and repression of immune responses. LincRNA-Cox2 bound hnRNP-A/B and hnRNP-A2/B1 in both the nuclear and the cytosolic compartments. These complexes regulated repression of immune genes, including Ccl5, but Il-6. It is unclear how lincRNA-Cox2 regulates Il-6 expression so far.

### linc1992/THRIL

Custom lincRNA microarray analysis of THP1-derived macrophages showed that Pam_3_CSK_4_ stimulation increased the levels of 127 lincRNAs and decreased the levels of 32 lincRNAs (25). In these lincRNAs, Pam_3_CSK_4_ stimulation decreased the expression of linc1992 (NR_110375) and knockdown of linc1992 notably restrained TNFα secretion. Linc 1992 is about 2 kb in length and widely expresses in human tissues. The authors also found other lncRNAs that regulates TNFα or IL6 secretion. Linc0206 (ENST00000450206, RP11-432J24), Linc7190 (ENST00000437190, RP11-296O14), and Linc7705(ENST00000417705, RP11-54O7) regulate TNFα and IL6 secretion under Pam_3_CSK_4_ stimulation. Moreover, Linc3995 (ENST00000523995, RP11-37B2) regulate IL6 secretion under Pam_3_CSK_4_ stimulation. However, it is unclear how these lncRNAs regulate cytokine expression.

Knockdown of linc 1992 decreased significantly production of TNFα mRNA. Thus, linc1992 regulates TNFα expression through a negative feedback system.

By the analysis of pull-down assay and RIP assay, linc1992 bound hnRNP-L and linc 1992 and hnRNPL formed an RNP complex *in vivo*. Moreover, ChIP assay revealed that hnRNPL bound to the TNFα promoter region and knockdown of linc1992 reduced binding of hnRNPL to the TNFα promoter region. Thus, this complex may regulate the transcription of downstream genes, including TNFα. Accordingly, Linc1992/THRIL (TNFα ανδ HnRNP-L Related Immunoregulatory LincRNA) is named after this regulation. Furthermore, Linc1992/THRIL regulated expression of IL-8, CXCL10, CCL1, and CSF1, but it is unclear whether hnRNP-L is involved in these expressions.

Moreover, linc1992/THRIL is associated with Kawasaki disease. Kawasaki disease is the most common cause of multisystem vasculitis in childhood. TNF-α is a pleiotropic inflammatory cytokine elevated during the acute phase of Kawasaki disease. In 17 patients with Kawasaki disease, linc1992/THRIL expression was lower during acute phase of disease when TNFα levels are elevated, so linc1992/THRIL could be a new biomarker for immune activation.

### lnc-IL7R

LncRNA microarray analysis showed that stimulation of THP-1 cells with lipopolysaccharide, a TLR4 ligand, induced the expression of 443 lncRNAs by more than twofold and decreased the expression 718 lncRNAs more than twofold. Among these lncRNAs, lnc-IL7R (ENST00000303115), one of the most up-regulated genes, is 1427 nt in length and overlaps the 3′-untranslated region (3′-UTR) of the human interleukin-7 receptor α-subunit (*IL7R*) (26). The majority of lnc-IL7R existed in the nucleus. The increase in lnc-IL7R expression after lipopolysaccharide stimulation indicates that lnc-IL7R is involved in the early immune response. Pam_3_CSK_4_ also increased lnc-IL7R expression, but poly I:C did not. In human peripheral blood mononuclear cells, stimulation by lipopolysaccharide or Pam_3_CSK_4_ increased lnc-IL7R levels. Lnc-IL7R negatively regulated E-selectin, VCAM-1, IL-8, and IL-6 expression following lipopolysaccharide stimulation. The mechanism by which lncRNA-IL7R regulated E-selectin and VCAM-1 was dependent on the trimethylation of histone H3 at lysine (H3K27me3). Lnc-1L7R knockdown also diminished IL-6 and IL-8 mRNA levels in an as yet unknown way.

## lncRNAs Involved in Bacterial Infection

### NeST/Tmevpg1

NeST, also known as Tmevpg1 or IFNG-AS1 (GS1-410F4), is an lncRNA located adjacent to the interferon (IFN)-γ-encoding gene in the *Tmevp3* locus that is a candidate gene in a susceptibility locus for Theiler’s virus (27, 28) in mouse. NeST RNA is encoded on the DNA strand opposite to that coding for IFN-γ, and the most abundant splice variant has six exons, 914 bp on length, in murine chromosome 10 and expressed in CD4^+^ T, CD8^+^ T, and natural killer cells.

By analysis of RIP and ChIP assay, NeST RNA bound WDR5, a subunit of the MLL/SET1 H3K4 methylase complex, and modified the chromatin at the *Ifng* locus. NeST RNA induced secretion of IFN-γ in CD8^+^ T cells. Transgenic mice, in which NeST is over-expressed, are protected against Theiler’s virus. NeST expression leaded to persistence of Theiler’s virus, but clearance of Salmonella infection.

### NEAT1

NEAT1 has two isoforms, 3.7 kb NEAT1v1 and 23 kb NEAT1v2 (29). NEAT1v2 is an essential factor in the structure of paraspeckles (30–33). Paraspeckles contain about 40 protein factors (34), including NONO/p54nrb and SFPQ/PSF, which function as a transcriptional inhibitor (35, 36).

NEAT1 was originally identified as an inducible lncRNA in mice brains infected with Japanese encephalitis or rabies viruses (37). HIV-I infection also induced NEAT1 in mice and NEAT1 modulates HIV-I replication by affecting the nucleus-to-cytoplasm export of Rev-dependent, instability element-containing HIV-I mRNAs (38). Moreover, it was found that poly I:C, a TLR3 ligand, herpes simplex virus 1 (HSV-1), and measles virus (MV) infection all induce NEAT1v2 (39). Furthermore, in normal cells, SFPQ, a paraspeckle protein, binds the IL-8 promoter to suppress its expression. Poly I:C stimulation, HSV-1, and MV infections caused the relocation of SFPQ from the *IL-8* gene promoter to NEAT1, resulting in the formation of excess paraspeckles, which in turn leaded to the transcriptional activation of *IL-8*.

## Conclusion

**Figure 1:**
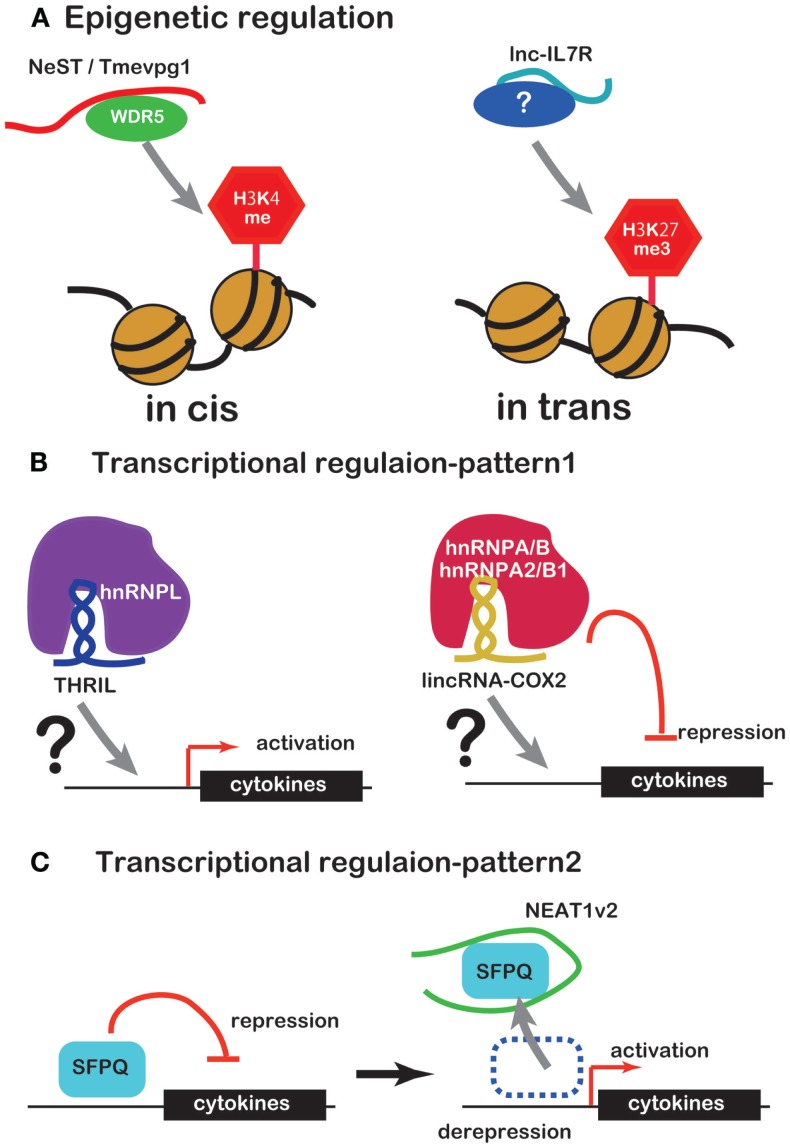
**ncRNAs that regulate chromatin structure and transcription factors**. **(A)** NeST/Tmevpg1 activates transcription of genes by recruiting the histone modifier WDR5, which methylates H3K4. Lnc-IL7R activates transcription of genes by H3K27 trimethylation. **(B)** The complex of THRIL and hnRNPL activates transcription of genes. The complex of lincRNA-Cox2 and hnRNPA/B or hnRNPA2/B1 represses transcription of genes. **(C)** NEAT1 activates transcription of genes by sequestration of transcriptional factor SFPQ.

Several lines of evidence have recently emerged concerning lncRNA involvement in the regulation of inflammatory mediator or cytokine expression. Unlike miRNAs, lncRNAs regulate expression of inflammatory mediators or cytokines by working with RBPs in a number of ways. Some complexes of lncRNAs and RBPs bind to promoters of inflammatory mediator or cytokine genes to regulate transcription or to modify the chromatin (Figures 1A,B). NEAT1 regulates the location of the transcription inhibitor SFPQ from promoter to paraspeckle (Figure 1C). These observations suggest that lncRNAs are factors of the innate immune response. However, there is only evidence that lncRNA induce under TLR ligand stimulation so far and little evidence that lncRNA function under the real bacterial infection, which induces more complicated event in cells. Further studies of this regulation by lncRNAs are likely to reveal novel drug targets for therapy of infectious and inflammatory diseases.

Why should ncRNAs be involved in regulating the expression of inflammatory mediators or cytokines? In virus-infected cells, translation is highly inhibited. For this reason, a translation-independent acute response system is required for an effective early immune response. Furthermore, involvement of ncRNA in such a response is biologically meaningful, because non-coding RNAs do not demand translational activity to express their functions. We therefore speculate that many other lncRNAs may play a role in situations where translation is highly repressed, such as viral infection, heat shock, and hypoxia.

## Conflict of Interest Statement

The authors declare that the research was conducted in the absence of any commercial or financial relationships that could be construed as a potential conflict of interest.
